# Analgesic Efficacy of Bupivacaine or Bupivacaine-Dexmedetomidine After Intraperitoneal Administration in Cats: A Randomized, Blinded, Clinical Trial

**DOI:** 10.3389/fvets.2019.00307

**Published:** 2019-09-13

**Authors:** Javier Benito, Marina C. Evangelista, Graeme M. Doodnaught, Ryota Watanabe, Guy Beauchamp, Beatriz P. Monteiro, Paulo Steagall

**Affiliations:** Department of Clinical Sciences, Faculty of Veterinary Medicine, Université de Montréal, Saint-Hyacinthe, QC, Canada

**Keywords:** analgesia, bupivacaine, dexmedetomidine, feline, intraperitoneal, ovariohysterectomy, pain

## Abstract

The aim of this study was to compare the analgesic efficacy of intraperitoneal bupivacaine vs. bupivacaine-dexmedetomidine in combination with intramuscular buprenorphine in cats undergoing ovariohysterectomy. Sixty healthy adult cats (2.8 ± 0.7 kg; *n* = 30/group) were included in a randomized, prospective, blinded, clinical trial after owners' written consent. After premedication with acepromazine (0.02 mg/kg) and buprenorphine (0.02 mg/kg) intramuscularly, anesthesia was induced with propofol to effect (6.2 ± 1.4 mg/kg) and maintained with isoflurane. Bupivacaine 0.25% alone (BG; 2 mg/kg) or bupivacaine (same dose) with dexmedetomidine (BDG; 1 μg/kg) were instilled/splashed over the ovarian pedicles and caudal aspect of uterus before ovariohysterectomy. Final injectate volume was standardized between groups. Sedation was evaluated using a five-point simple descriptive scale. Pain was evaluated using the short-form UNESP-Botucatu composite pain scale (SF-CPS) before, and at 0.5, 1, 2, 3, 4, 6, 8, 12, and 24 h after surgery. Rescue analgesia was provided with buprenorphine (0.02 mg/kg intravenously) and meloxicam (0.2 mg/kg subcutaneously) when SF-CPS ≥ 4. The Mantel-Haenszel chi-square test was used for analyzing ordinal variables (e.g., SF-CPS pain scores). The effect of time in SF-CPS scores was assessed with the Cochran-Mantel-Haenszel test for repeated measures. The alpha level for each contrast was adjusted downward with the sequential Benjamini-Hochberg procedure. The number of cats receiving rescue analgesia was analyzed using χ2 test (*p* < 0.05). The prevalence of rescue analgesia was the same for the two treatments (*p* = 1.000) [BG, *n* = 6, 20%; BDG, *n* = 6, 20%] and similar for timing of rescue analgesia (*p* = 0.16). The SF-CPS scores were significantly increased between 1 and 12 h in BG, and between 0.5 and 8 h in BDG when compared with baseline values. Median (interquartile range) pain scores were higher in BG [1 (1–2)] than BDG [1 (0–1)] at 12 h (*p* = 0.023). Sedation scores were not significantly different between groups throughout the study. In terms of prevalence of rescue analgesia, but not duration of action, the analgesic efficacy of bupivacaine-dexmedetomidine was similar to bupivacaine alone after intraperitoneal administration in cats receiving buprenorphine.

## Introduction

The World Small Animal Veterinary Association Global Pain Council has recommended the use of incisional and intraperitoneal administration of local anesthetics for canine and feline pain management in a multimodal analgesic approach. These are simple, safe and cost-effective methods to reduce pain after ovariohysterectomy in small animals (1).

Local anesthetic drugs are often combined with adjuvant drugs (i.e., opioids, vasoactive agents, agonists of α-adrenergic receptors, etc.) in human and veterinary medicine to prolong analgesia after peripheral nerve blocks (2, 3). This combination has also been used in clinical trials using the intraperitoneal technique in cats and humans (4–6). Dexmedetomidine is an agonist of α-2 adrenergic receptors with sympatholytic, sedative, and analgesic effects. In cats, dexmedetomidine has been used in combination with bupivacaine to improve antinociception and duration of action after sciatic and femoral nerve blocks (3).

The pharmacokinetics, safety and efficacy of bupivacaine after intraperitoneal administration have been demonstrated in cats (6, 7). Plasma concentrations of bupivacaine after intraperitoneal administration were below toxic levels in eight cats (6), and no clinically detectable adverse-effects were observed in a subsequent clinical trial involving 45 cats (7). In the latter study, the technique was shown to provide analgesia in cats being administered buprenorphine preoperatively and the prevalence of rescue analgesia in the latter group was similar to cats receiving buprenorphine and meloxicam (7). Furthermore, the pharmacokinetics and analgesic effects of intraperitoneal administration of bupivacaine in combination with dexmedetomidine or epinephrine was evaluated in 16 cats undergoing ovariohysterectomy and receiving meloxicam preoperatively (8). Plasma concentrations were below toxic levels and no adverse-effects were observed. The analgesic efficacy was similar between the two treatments (8). However, the efficacy of bupivacaine alone in comparison with bupivacaine-dexmedetomidine after intraperitoneal administration has not been studied in a large prospective, randomized trial, and it is not known if there is an advantage of using bupivacaine-dexmedetomidine over bupivacaine alone to extend the magnitude and duration of postoperative analgesia.

The aim of this study was to compare the analgesic efficacy of intraperitoneal bupivacaine vs. bupivacaine-dexmedetomidine in cats undergoing ovariohysterectomy and receiving buprenorphine. The hypothesis was that the pain scores and the prevalence of rescue analgesia would be lower after bupivacaine-dexmedetomidine than bupivacaine alone in cats receiving buprenorphine.

## Materials and Methods

The study protocol was approved by the local animal care committee, *Comité d'éthique de l'utilisation des animaux (Université de Montréal*) (18-Rech-1825).

### Animals

Eighty-one mixed-breed adult female cats (2.8 ± 0.7 kg) from three local animal shelters were admitted to the veterinary teaching hospital (*Center Hospitalier Universitaire Vétérinaire*) of the Faculty of Veterinary Medicine, Université de Montréal for elective ovariohysterectomy between June and October 2018. Written consent for participation in the study was obtained for each patient.

Cats were included if they were considered healthy based on history, complete physical examination and had values of hematocrit and total protein within reference range. Exclusion criteria included body weight <1 kg, cardiac arrhythmias, pregnancy, lactation, body condition score >7 or <3 on a scale from 1 to 9, anemia (hematocrit < 25%), hypoproteinemia (total protein < 59 g/L), baseline pain scores ≥3 or clinical signs of systemic disease. Shy or fearful individuals were also excluded. Cats were housed individually in adjacent cages in a cat ward. Each cage was equipped with water and food bowls and a litter box. Environmental enrichment included a hanging toy, blankets and a box where the cat could hide inside or use as an elevated surface. This study is reported according to the CONSORT guidelines for randomized, clinical trials (9).

### Experimental Design and Treatment Groups

This study was a prospective, randomized, blinded, clinical trial. Upon arrival, each cat was sequentially assigned a tentative number (1–60). This number was only confirmed if the cat was finally included in the trial. Therefore, the number of an excluded cat became once again available to another cat until 60 cats were included.

Randomization sequence^1^ was created with a 1:1 allocation of two blocks of 32 individuals. Cats received one of the following two treatments by the intraperitoneal route of administration: bupivacaine (0.25% Bupivacaine Injection BP, SteriMax Inc., Canada) 2 mg/kg (BG, bupivacaine group) or bupivacaine (same dose) and dexmedetomidine (Dexdomitor®, Zoetis Canada Inc., Canada) 1 μg/kg (BDG; bupivacaine-dexmedetomidine group) according to previous publications in our laboratory (6–8). The bupivacaine-dexmedetomidine solution was prepared by adding 0.025 mL of dexmedetomidine (0.5 mg/mL) into a 10-mL vial of bupivacaine. The volume of dexmedetomidine was withdrawn using an insulin syringe of 0.3 mL. Final injectate volumes (0.8 mL/kg) were equal for both groups. Individuals involved with treatment administration were not involved with pain assessment. Therefore, the observer scoring pain was blinded to treatment.

### Anesthetic and Surgical Procedures

Food but not water was withheld for 8–12 h before general anesthesia. Cats were pre-medicated with acepromazine (0.02 mg/kg; Atravet, Boehringer Ingelheim, Canada) and buprenorphine (0.02 mg/kg; Vetergesic, Champion Alstoe Animal Health, Canada) intramuscularly. Approximately 20 min before induction, a 22-G catheter was inserted aseptically into a cephalic vein. Anesthesia was induced using propofol (PropoFlo 28, Zoetis, Canada) intravenously to effect. One mg of lidocaine 2% (Lurocaine, Vetoquinol, Canada) was instilled over the arytenoid cartilages before endotracheal intubation with an appropriately sized, cuffed endotracheal tube. All cats were connected to a small animal anesthesia machine using a Mapleson D system. Anesthesia was maintained with isoflurane (Isoflurane USP, Fresenius Kabi, Canada) delivered in oxygen. Anesthesia was performed by the same veterinarian (RW). Cats were positioned in dorsal recumbency on a circulating warm water blanket and monitored with a multiparametric monitor (Lifewindow 6000V, Digicare Animal Health, USA). Continuous monitoring consisted of electrocardiogram, capnography, non-invasive blood pressure, and pulse oximetry which were recorded every 5 min throughout the procedure. Lactated Ringer's solution (Lactated Ringer's solution USP, Baxter, Canada) was administered intravenously at a rate of 10 mL/kg/h during anesthesia. The intraperitoneal solution (BG or BDG) was withdrawn aseptically by the surgeon using a 3 or 5-mL syringe attached to a 22-gauge needle. The needle was removed, and the solution instilled (i.e., splashed) over the right and left ovarian pedicles, and the caudal aspect of the uterine body in three equal volumes immediately after the midline incision of the skin, subcutaneous tissues, and linea alba. Ovariohysterectomy was always performed by the same veterinarian (BM) ~1 min after intraperitoneal administration. Surgery was performed using the pedicle tie technique as previously described (10). The body wall and the skin were closed using simple continuous and intradermal suture patterns, respectively. At the end of the surgical procedure, a 2-cm green line tattoo was applied lateral to the ventral midline incision for visual identification of a neutered animal. Duration of surgery (time elapsed from the first incision to last suture), anesthesia (time elapsed from beginning to cessation of isoflurane administration), and time to extubation (time elapsed from cessation of isoflurane administration until extubation) were recorded for each cat.

### Outcomes: Pain and Sedation Scoring, and Prevalence of Rescue Analgesia

Pain was evaluated by one observer (ME) who was not aware of treatment groups using the short-form UNESP-Botucatu composite pain scale (SF-CPS), a novel pain scoring system that has undergone thorough validation using video assessment (Steagall P, personal communication). The SF-CPS consists of four items (0–3 points for each item) to evaluate the cats' posture, activity, attitude and reaction to touch and palpation of a painful site with a maximum total score of 12. Rescue analgesia was provided with buprenorphine 0.02 mg/kg intravenously and meloxicam (Metacam, Boehringer Ingelheim, Canada) 0.2 mg/kg subcutaneously when SF-CPS ≥4. Pain assessments were performed at 60 min before induction of anesthesia (time 0; baseline) and at 0.5, 1, 2, 3, 4, 6, 8, 12, and 24 h following the end of surgery. Additional rescue analgesia was administered if needed with the same dose of buprenorphine. If a cat did not require rescue analgesia until the 12 h evaluation time-point, meloxicam was administered (same dose and route of administration as described above). Data collected after the administration of rescue analgesia were not included in the statistical analysis.

Sedation was assessed with a simple descriptive five-point scale, where a score of 0 = no sedation; 1 = cat was able to stand but is wobbly; 2 = in sternal recumbency; 3 = can lift its head; 4 = fast asleep/no response to hand clap (11).

### Sample Size and Statistical Analysis

A priori power analyses were performed with an online calculator^2^ The authors considered the mean pain scores of 3.6 and 2.9 from previous publications using IP bupivacaine (7) and bupivacaine with dexmedetomidine (8), respectively. Considering the mean difference in pain scores between treatments of 0.7 points and the predefined standard deviation of ±1 point, a sample size of 32 animals per group would provide power of 80% at the α level of 5%.

Statistical analyses were performed with SAS (version 9.3; SAS Institute, USA) and figures were plotted with GraphPad Prism 8 (version 8.0.2; GraphPad Software Inc, USA). Demographic data for each group were analyzed using equal variances *t*-tests. Sedation scores between groups were compared with Mann-Whitney U test, and the effect of time on sedation scores was assessed using Friedman test and Dunn's Multiple comparison tests. The alpha level was adjusted using the Bonferroni correction. The SF-CPS scores between groups were compared with the Mantel-Haenszel chi-square test for ordinal variables. The effect of time on SF-CPS scores was assessed with the Cochran-Mantel-Haenszel test for ordinal variables and repeated measures. The alpha level for each contrast was adjusted downward with the sequential Benjamini-Hochberg procedure. The number of cats receiving rescue analgesia was analyzed within their treatment group using the chi-square test. Timing for rescue analgesia was analyzed with Wilcoxon test. Values of *p* < 0.05 were considered significant.

## Results

### Demographic Data

**Figure 1:**
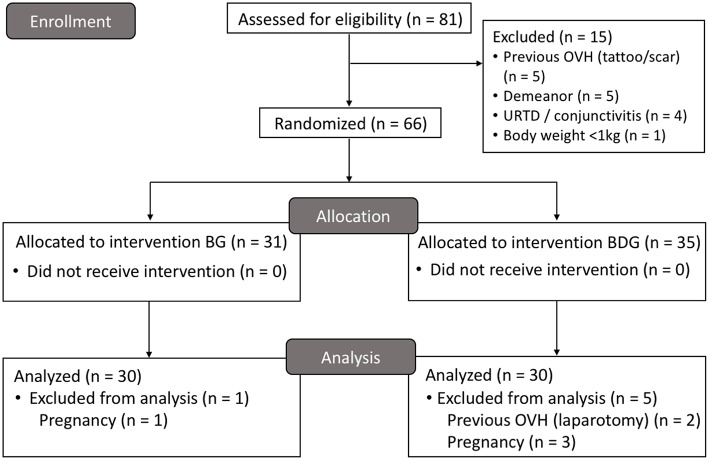
CONSORT flow diagram. BG, intraperitoneal bupivacaine (bupivacaine 0.25%, 2 mg/kg); BDG, intraperitoneal bupivacaine (bupivacaine 0.25%, 2 mg/kg) and dexmedetomidine (1 μg/kg); OVH, ovariohysterectomy; URTD, upper respiratory tract disease.

Sixty cats were included, and 21 cats were excluded in the study. The high number of excluded cats was unexpected, and the study was terminated when 30 cats per group were reached due to time and budget constraints. Figure 1 shows the number of included and excluded animals using the CONSORT flow diagram. Table 1 shows initial body condition score, body weight, age, hematocrit and total protein, duration of anesthesia and surgery, and time to extubation (*p* > 0.05). Mean ± SD dose of propofol was 6.2 ± 1.4 mg/kg (5.9 ± 1.0 mg/kg for BG and 6.5 ± 1.6 mg/kg for BDG, *p* = 0.0686). All cats were discharged 24 h after surgery without postoperative complications.

**Table 1:** Demographic data, values of hematocrit and total protein, duration of anesthesia and surgery, and time to extubation.

**Variables**	**All cats** ***n* = 60**	**BG** ***n* = 30**	**BDG** ***n* = 30**	***p*-value**
**Body condition scores (1–9)**	4 (3–6)	4 (3–6)	4 (3–5)	0.1964
**Body weight (kg)**	2.8 ± 0.7	2.92 ± 0.65	2.66 ± 0.69	0.1359
**Age (years)**	1.36 ± 0.87	1.46 ± 0.86	1.25 ± 0.88	0.4054
**Hematocrit (%)** Reference range (28–47)	33 ± 5	33 ± 5	33 ± 4	1
**Total protein (g/L)** Reference range (59–81)	67 ± 7	68 ± 7	66 ± 7	0.1351
**Duration of anesthesia (min)**	34 ± 5	36 ± 7	33 ± 4	0.0580
**Duration of surgery (min)**	20 ± 2	21 ± 3	20 ± 2	0.1173
**Time to extubation (min)**	2 ± 1	2 ± 1	2 ± 1	0.1036

*Body condition score, body weight, age, hematocrit and total protein, duration of anesthesia and surgery, and time to extubation in cats undergoing ovariohysterectomy after intraperitoneal administration of bupivacaine alone (BG) or bupivacaine-dexmedetomidine (BDG). Data presented as median (range) or mean ± SD where applicable. Values of p <0.05 were considered significant*.

### Pain Scores

**Figure 2:**
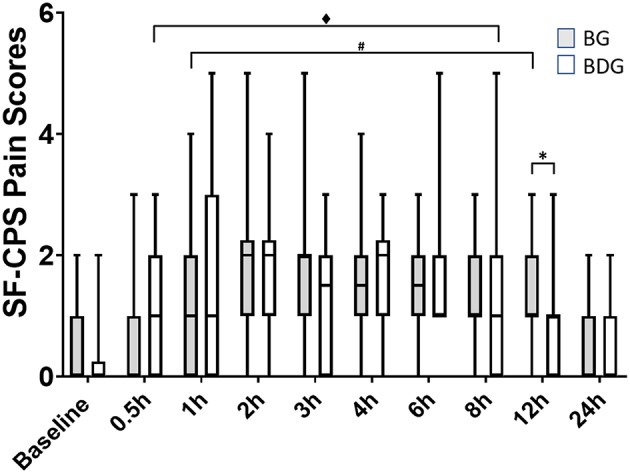
Pain scores. Boxplots showing the pain scores with median, interquartile range and minimum and maximum values, using the short form of the multidimensional composite pain scale (UNESP-Botucatu; SF-CPS). Pain scores correspond to a 12-point scale, where the cut-off score for administration of rescue analgesia was ≥4. BG, intraperitoneal bupivacaine (bupivacaine 0.25%, 2 mg/kg); BDG, intraperitoneal bupivacaine (bupivacaine 0.25%, 2 mg/kg) and dexmedetomidine (1 μg/kg). All cats received meloxicam (0.2 mg/kg SC) at 12 h postoperatively. Values of *p* < 0.05 were considered significant. *Significant difference between groups. ^#^Significant difference when compared with baseline (BG). ^♦^Significant difference when compared with baseline (BDG).

Median (interquartile range) SF-CPS scores were higher in BG [1 (1–2)] than in BDG [1 (0–1)] at 12 h (*p* = 0.023) (Figure 2), but not at other times. When compared with baseline, the SF-CPS scores were increased in BG at 1 h (*p* = 0.0019), 2 h (*p* = 0.0002), 3 h (*p* = 0.001), 4 h (*p* = 0.0005), 6 h (*p* = 0.0006), 8 h (*p* = 0.002) and 12 h (*p* = 0.0038), but not at 30 min (*p* = 0.29) and 24 h (*p* = 0.56) and in BDG at 30 min (*p* = 0.0097), 1 h (*p* = 0.007), 2 h (*p* < 0.0001), 3 h (*p* = 0.002), 4 h (*p* < 0.0001), 6 h (*p* = 0.0001), and 8 h (*p* = 0.008), but not at 12 h (*p* = 0.33) and 24 h (*p* = 0.29) (Figure 2).

### Prevalence of Rescue Analgesia

The number of cats requiring rescue analgesia was the same for BG and BDG (*p* = 1.000; [BG, *n* = 6, 20%; BDG, *n* = 6, 20%]). Timing for rescue analgesia were as follows: BG; at 1 h (*n* = 1), 2 h (*n* = 3), 3 h (*n* = 1), and 4 h (*n* = 1) post-operatively, and BDG; at 1 h (*n* = 1), 2 h (*n* = 1), 6 h (*n* = 2) and 8 h (*n* = 2) postoperatively. The timing for rescue analgesia did not differ between treatments (*p* = 0.16).

### Sedation Scores

Median (interquartile range) sedation scores were as follows: BG [baseline, 0 (0–0); 0.5 h, 1 (1–1); 1 h, 1 (0–2); 2 h, 1 (0–2); 3 h, 0 (0–2); 4 h, 0 (0–2); 6 h, 0 (0–2); 8 h, 0 (0–0); 12 h, 0 (0–0); 24 h, 0 (0–0)] and BDG [baseline, 0 (0–0); 0.5 h, 1 (1–2); 1 h, 0 (0–1); 2 h, 1 (0–2); 3 h, 0 (0–2); 4 h, 0 (0–2); 6 h, 0 (0–0); 8 h, 0 (0–2); 12 h, 0 (0–0); 24 h, 0 (0–0)]. Sedation scores were not significantly different between groups at any time point (*p* > 0.999). The sedation scores were increased at 0.5 h (*p* < 0.001) and no significant differences were found at 1 h (*p* > 0.99), 2 h (*p* = 0.392), 3 h (*p* > 0.999), 4 h (*p* > 0.999), 6 h (*p* > 0.999), 8 h (*p* > 0.999), 12 h (*p* > 0.999), and 24 h (*p* > 0.999) when compared with baseline.

## Discussion

In terms of prevalence of rescue analgesia, the analgesic efficacy following intraperitoneal administration of bupivacaine-dexmedetomidine was similar to bupivacaine alone in cats undergoing ovariohysterectomy receiving buprenorphine. Based on the prevalence and timing of rescue analgesia, the study could not show an advantage of using bupivacaine-dexmedetomidine over bupivacaine alone. However, pain scores were lower in BDG than in BG cats at 12 h, and these scores returned to baseline values before in BDG (8 h) than in BG (12 h) cats suggesting a prolonged analgesic effect with BDG. This finding is in agreement with our previous pharmacokinetic studies. The intraperitoneal administration of bupivacaine-dexmedetomidine delayed time to peak plasma concentrations (123 ± 59 min) (8) when compared with bupivacaine alone (30 ± 24 min) (6). Therefore, it could be argued that the local vasoconstriction produced by dexmedetomidine played a role in delayed absorption and longer terminal elimination half-life, and increased duration of effect in BDG. However, it is difficult to predict if these findings are clinically relevant considering the mean pain scores for each group at the 12 h-time point. In addition, no cats received rescue analgesia at 12 h. In other words, some cats in BG could be slightly more painful than cats in BDG without the need of further administration of analgesics. It is also possible that this significant difference may have occurred simply by chance given the large number of comparisons performed in our study, despite adjustment for multiple comparisons.

The prevalence of rescue analgesia was 20% in this study even when using a combination of an opioid (i.e., buprenorphine) with a local anesthetic technique. This prevalence could have been even lower if a non-steroidal anti-inflammatory drug (NSAID) had been administered as part of multimodal analgesia as reported in our previous study when none of the cats required rescue analgesia (8). Indeed, the combination of local anesthetic blocks (i.e., intraperitoneal anesthesia), buprenorphine and a NSAID is commonly used in our clinical practice for feline analgesia. However, the administration of a NSAID could have masked the effects of intraperitoneal bupivacaine alone or in combination with dexmedetomidine, and intramuscular buprenorphine in this study, and biased the results of this study. Hence, the authors decided to administer meloxicam 12 h after surgery to ensure comfort of these patients overnight, if cats had not been given rescue analgesia.

In a similar clinical trial, dogs undergoing ovariohysterectomy received a full agonist of μ-opioid receptors (morphine), preoperative NSAID and intraperitoneal bupivacaine or ropivacaine. The prevalence of rescue analgesia was higher than in the present study (27% for bupivacaine and 40% for ropivacaine) (12). However, surgery was performed by veterinary students and duration of anesthesia was much longer than in our study which could explain these differences. The same experienced veterinarian performed the surgeries in the study herein and in the aforementioned studies in cats in our laboratory with the goal of providing similar and consistent level of tissue manipulation/damage and size of incision during celiotomy (6–8).

There were limitations in this study. Pain was evaluated using the SF-CPS; this novel pain scoring system based on the UNESP-Botucatu multidimensional pain scale. The SF-CPS has been validated using video, but not real-time assessment (Steagall P, personal communication). Future publications on the use of the SF-CPS will corroborate the validity of this instrument as robust means of feline pain assessment. A control group was not included in this study. Considering that previous reports support the superior analgesic efficacy of the combination buprenorphine-intraperitoneal bupivacaine over buprenorphine-intraperitoneal saline (7) and the potential ethical concerns with repeating similar research, the addition of a control group was considered unnecessary. Meloxicam was administered at 12 h after surgery which precluded further conclusions on the analgesic efficacy between BDG and BG afterwards. Indeed, most cats usually require rescue analgesia up to 6 h after surgery and differences between groups were generally less evident after this period in previous studies in cats undergoing ovariohysterectomy (7, 13). Thus, decreases in pain scores over time were also likely to mask differences between groups. Based on the pharmacological profile of agonists of α_2_-adrenergic receptors, it is possible that the BDG could produce better analgesia than BG at later time points (e.g., 16 h) considering the ability of these drugs to produce local vasoconstriction and prolong the anesthetic effect of local anesthetics as demonstrated in humans (14–18). Hence, the lack of difference between groups at 24 h should be interpreted with caution because the administration of meloxicam might have confounded these results. However, in the authors' experience, most cats require rescue analgesia in the early postoperative period (i.e., up to 8 h after surgery) after ovariohysterectomy (6, 8, 13). The efficacy of intraperitoneal analgesia may depend on doses, concentrations, volumes of injection, mode of administration (i.e., aerosol, nebulization vs, instillation), and the addition of adjuvant drugs (8). Therefore, for example, it is not known how higher doses and lower concentrations of dexmedetomidine in combination with bupivacaine would have influenced the pharmacokinetic and pharmacodynamic profile of bupivacaine-dexmedetomidine in comparison with bupivacaine alone. Another limitation is the lack of another group of cats receiving the intraperitoneal technique alone (i.e., without buprenorphine). However, the intraperitoneal technique has been recommended for pain management as part of a multimodal analgesic approach (1) and the aim of the study was to compare the efficacy of BG vs. BDG and not a multimodal (i.e., buprenorphine and intraperitoneal administration of local anesthetics) vs. a unimodal technique (i.e., intraperitoneal administration of local anesthetics alone) in this study. Future research is warranted to investigate other aspects of the intraperitoneal administration of local anesthetics in feline practice including different adjuvants, doses, and techniques.

## Conclusions

In terms of prevalence of rescue analgesia, but not duration of action, the analgesic effects after the intraperitoneal administration of bupivacaine-dexmedetomidine were similar to bupivacaine alone in cats undergoing ovariohysterectomy receiving buprenorphine. The administration of a NSAID, buprenorphine and intraperitoneal bupivacaine could reduce the prevalence of rescue analgesia observed in this study.

## Data Availability

The datasets generated for this study are available on request to the corresponding author.

## Ethics Statement

The animal study was reviewed and approved by Comité d'éthique de l'utilisation des animaux (Université de Montréal) (18-Rech-1825). Written informed consent was obtained from the owners for the participation of their animals in this study.

## Author Contributions

JB, ME, and PS designed and conducted the study, and drafted the manuscript. JB and ME performed the postoperative pain assessments and helped with data analyses. GD conducted the study. RW performed general anesthesia. GB performed the statistical analysis and helped with data interpretation. BM performed the surgeries and postoperative care. All authors reviewed and approved the final manuscript.

### Conflict of Interest Statement

PS has provided consultancy services and has received speaker honoraria from Zoetis. BM has provided consultancy services in pharmacovigilance to Zoetis. The remaining authors declare that the research was conducted in the absence of any commercial or financial relationships that could be construed as a potential conflict of interest.

## Footnotes

^1^Randomization plan generator available from: https://www.randomization.com

^2^Sample size calculator available from: https://clincalc.com/stats/samplesize.aspx
